# Detection of resistance to macrolides and fluoroquinolones in *Mycoplasma genitalium* by targeted next-generation sequencing

**DOI:** 10.1128/spectrum.03845-23

**Published:** 2024-02-13

**Authors:** Calin B. Chiribau, Sarah Schmedes, Yibo Dong, Namratha Tarigopula, Omer Tekin, Andrew Cannons, Jill Roberts, Donna Haiduven, Susanne R. Crowe

**Affiliations:** 1 Florida Department of Health, Bureau of Public Health Laboratories, Jacksonville, Florida, USA; 2 University of South Florida, College of Public Health, Tampa, Florida, USA; University of Texas Southwestern Medical Center, Dallas, Texas, USA

**Keywords:** *Mycoplasma genitalium*, resistance, sexually transmitted infection, treatment failure, macrolide resistance, fluoroquinolone resistance, resistance-associated mutations

## Abstract

*Mycoplasma genitalium* is fastidious to culture, and its detection in human clinical specimens relies mainly on molecular methods. Phenotypic determination of antibiotic susceptibility for this bacterium is not a timely or feasible option for most clinical laboratories. This study sought to determine whether next-generation sequencing technologies can effectively be employed in determining genetic mutations associated with drug resistance in *M. genitalium* samples collected in Aptima Hologic tubes and possibly integrating them into viable workflows in public health laboratories. Following analysis by a custom-designed bioinformatics pipeline, at least one mutation/sample has been identified in 94/98 specimens in at least one of seven loci (macrolides: *rrl*, *rplD*, *rplV*; fluoroquinolones: *parC*, *parE*, *gyrA*, *gyrB*) described previously to be connected to antibiotic resistance. This method identified a total of 469 single nucleotide polymorphisms (SNPs) (452 mutations): 134 of 23S rRNA SNPs and 318 amino acid mutations: 114 substitutions and 204 synonymous; the turnaround time (sample to analyzed sequence) was typically 3 days. The assays and workflows described in this work demonstrated that the determination of a drug resistance profile for macrolides and fluoroquinolones of *M. genitalium* samples by using next-generation sequencing in clinical samples is a feasible approach that can be implemented in clinical laboratories, following thorough and extensive validation studies.

**IMPORTANCE:**

The mechanisms of drug resistance in *Mycoplasma genitalium* are complex and involve several genetic loci. The molecular methods for accurately characterizing resistance to fluoroquinolones and macrolides in this organism are often not available or approved for patient use and do not cover all genetic determinants. To this end, we propose a next-generation sequencing-based method with a turnaround time of 3 days that includes the investigation of all drug resistance loci of *M. genitalium*. Following adaptation, validation, and verification for routine clinical use, assays based on this method may yield molecular results that can be used to guide proper treatment regimens and for surveillance of drug resistance in the general population.

## INTRODUCTION

The mollicute *Mycoplasma genitalium* is a slow-growing, sexually transmitted bacterium, notoriously difficult to culture and characterize (1). It usually infects the mucous epithelia of the human urinary and genital tract, and due to the absence of a cell wall, it is naturally resistant to antibiotics targeting this cellular component, such as β-lactams. Infections with this microorganism are often asymptomatic, but its importance in sexual health has been gaining more recognition in recent years. They are now recognized as a cause of non-gonococcal urethritis in males, and cervicitis, pelvic inflammatory disease in females. *M. genitalium* has been linked to infertility and an increased risk of preterm birth and miscarriages (2), but other studies reported no such associations (3, 4).

While employing proper antibiotics is of paramount importance for a successful treatment regimen, due to its fastidiousness and slow-growing characteristics, determination of a susceptibility profile by phenotypical antimicrobial susceptibility testing is not a timely and viable option. In addition, resistance to two main classes of antimicrobials (macrolides and fluoroquinolones) has been documented as early as 2010 (5, 6).

Interestingly, even though resistance mechanisms for tetracyclines have not been clearly defined in *M. genitalium* so far, doxycycline treatment failures occur in >50% of the cases (7). For other antibiotics, resistance has been linked to *M. genitalium* chromosomal mutations and may lead to treatment failures (8).

The 2021 Centers for Disease Control and Prevention STI guidelines for cases where azithromycin (macrolide) resistance testing is not available recommend doxycycline for seven days, followed by moxifloxacin (fluoroquinolone) for another week (9). The effectiveness of a resistance-guided sequential treatment approach that includes doxycycline and azithromycin (if macrolide resistance mutations [MRMs] are not present) or doxycycline and moxifloxacin (if MRMs are present) has been demonstrated (10) and is now included in the 2021 CDC STI guidelines.

Although the need is evident, commercial tests to screen for antimicrobial resistance to macrolides or fluoroquinolones in *M. genitalium* are not yet available or approved by the Food and Drug Administration in the United States (1).

This study has evaluated the utility of using amplicon-based next-generation sequencing to assess the drug resistance profile (macrolides and fluoroquinolones) of *M. genitalium*-positive samples collected in Hologic transport media. While “classical” DNA-sequencing technologies (e.g., Sanger) have been used extensively, this is to the best of our knowledge only the second study (11) to make use of targeted next generation sequencing (NGS) in determining drug resistance in *M. genitalium*.

The authors of this study (11) assessed the performance of a custom amplicon sequencing approach in *n* = 52 *M*. *genitalium*-positive samples of cervical, vaginal, anal and rectal swabs, and urine origins. Three antibiotic resistance loci (23S rRNA, *parC,* and *gyrA*) were targeted, and single nucleotide polymorphisms (SNPs) were detected in 94%, 56%, and 4% of the samples, respectively. The method uses an ingenious dual index primer design to amplify individual loci (thus eliminating the library preparation step), and it is scalable and arguably superior to classical methods as it can determine low-frequency alleles usually not detected by Sanger sequencing.

Our work aimed primarily to explore a NGS-based protocol that may be adapted in clinical laboratories that perform high-complexity testing (i.e., public health laboratories) and to determine a complete molecular characterization of the genetic drug resistance determinants for macrolides and fluoroquinolones. Amplification and sequencing of the seven loci described so far in the literature as being associated with resistance have been attempted for all specimens included in the study. The samples used were remnants of Aptima Hologic tubes used for the detection of *Chlamydia, Neisseria gonorrhea,* and *M. genitalium*, and thus a workflow based on reflex testing on the basis of detection positivity may be envisioned.

## MATERIALS AND METHODS

### Experimental design and samples

Randomly selected, de-identified, and remnant samples collected in Hologic transport media from the state of Florida from 2021–2023, which were submitted to the Florida Bureau of Public Health Laboratories (FBPHL) for routine gonorrhea and chlamydia testing, were also screened for the detection of *M. genitalium*. The study protocol was reviewed by the Institutional Review Boards (IRBs) of the Florida Department of Health and the University of South Florida, and both IRBs determined that the study did not constitute research involving human subjects, which was in accordance with DHHS and FDA regulations. The initial samples were collected from both self-identified males and females, and included various specimen types: urine, vaginal swabs, cervical swabs and rectal swabs. After screening, remnant *M. genitalium*-positive samples were stored at −20°C until testing.

The genomic regions investigated using sequencing covered 4,461 bp, and they were as follows: *rrl* (23S rRNA: nucleotides 1985–2682), *rpl*D (L4, entire ORF), *rpl*V (L22, entire ORF), *gyr*A (amino acids 47–146), *gyr*B (amino acids 427–549), *par*C (entire ORF), and *parE* (amino acids 119–509). All these genetic regions were previously described to be associated with antibiotic resistance in this microorganism (12, 13, 14).

### Nucleic acid extraction

Before processing, the samples were allowed to thaw at room temperature. Urine samples were incubated in a water bath at 37°C for 5 minutes to dissolve crystals (that may impair extraction) as described in (15). Nucleic acid extraction was performed using the MagMAXViral/Pathogen Nucleic Acid Kit and the KingFisher Flex Magnetic Particle Processor instrument with 96 Deep-Well Head (ThermoFisher Scientific, Waltham, MA, USA). A typical sample extraction batch contained 24 samples. In short, one extraction reaction contained 10 µL proteinase K solution, 400 µL sample, 530 µL binding solution, and 20 µL total nucleic acid beads. Extraction on the KingFisher Flex instrument was done following the MVP_2Wash_400_Flex protocol, and samples were eluted in 50 µL elution buffer. After completion of the protocol, the eluted samples were immediately removed from the instrument and stored at −20°C.

### PCR amplification

PCR amplifications of the seven loci were done using the primers listed in Table 1, procured from IDT DNA (Coralville, IA, USA).

A partial multiplex PCR approach was designed for this study, and four PCRs (final volume 25 µL) were used per sample: reaction 1: *rrl*; reaction 2: *rplD, gyrB*; reaction 3: *gyrA, parE*; and reaction 4: *rplV, parC*. A PCR consisted of 7 µL DNase-free water, 12.5 µL Platinum Superfi II 2× mix (Invitrogen), 2.5 µL primer mix (each primer at a final concentration of 0.5 µM), and 3 µL template (DNA extracts). The cycling program was as follows: 98°C for 30 seconds, 98°C for 10 seconds, 60°C for 10 seconds, 72°C for 30 seconds (steps 2–4: 45 cycles), 72°C for 5 minutes, and 10°C hold. Each PCR run included the *M. genitalium* reference strain G37 genomic DNA (ATCC #33530D; ATCC, Manassas, VA, USA) as a positive control. The G37 gDNA was diluted 10× before amplification, and the run was valid only if all seven amplicons were amplified in this sample. This partial multiplex approach helped conserve reagents and allowed the amplification of 24 clinical samples on a 96-well PCR plate.

**TABLE 1:** DNA primers used in this study

Locus	Primers	Genomic position (G37-type strain)	Sequence (5′–>3′)	PCR (bp)
*rrl* (23S rRNA)	23S rRNA-F	173713 to 173738	GTGTAACCATCTCTTGACTGTCTCGG	696
	23S rRNA-R	174385 to 174409	CGGTCCTCTCGTACTAGAAGCAAAG	
*rplD* (L4)	L4 -F	191138 to 191164	AAGTAATGGCTAAACTTAAAGTAATCC	669
	L4-R	191780 to 191807	TTTAAGAGTATGTTGGTTACATCCATAG	
*rplV* (L22)	L22-F	193154 to 193180	ATGGTAGGTCATAAGTTGGGTGAGTTT	559
	L22-R	193686 to 193713	AGTTCTTATTAATGCCAAACCTTAAGCC	
*gyr*A	*gyr*A-F	4950 to 4973	CCTGATGCTAGAGATGGACTTAAA	299
	*gyr*A-R	5225 to 5249	AAGTTCTGCTGCAAGTTTAGATAAT	
*gyr*B	*gyr*B-F	4122 to 4143	TTGTACAACCAGAGATCCTTCG	370
	*gyr*B-R	4473 to 4492	GTGGGGGTTGAGCAATAAAA	
*par*C	*par*C-F	241832 to 241850	GGCGCACACATCCAAATCC	711
	*par*C-R	242521 to 242543	CCATGGATAGAAACAGTTGTTCA	
*par*E	*par*E-F	240675 to 240694	GTGCATCAGTGGTCAATGCC	1157
	*par*E-R	241832 to 241850	GGATTTGGATGTGTGCGCC	

### Gel electrophoresis

PCR products for each sample were assessed using gel electrophoresis (E-Gels double-comb agarose gels with SYBR safe NDA gel stain 2%; Invitrogen). Samples that yielded satisfactory amplicons (DNA fragments of the lengths indicated in Table 1) for all seven PCR reactions and a small set of samples with an incomplete amplification panel were selected.

### Next-generation sequencing

Amplicon pooling and purification: a typical tNGS run for this assay included 32 samples; 10 µL of each of the PCRs belonging to a sample were pooled (total volume: 40 µL), mixed with an equal volume of AxyPrep MagPCR cleanup beads (Corning, Glendale, AZ, USA), purified by two 80% ethanol washes, and eluted in 50 µL DNase-free water. DNA concentrations of purified amplicon pools were determined using Qubit BR (ThermoFisher Scientific).

Library amplification and purification: 1 ng of each purified amplicon pool was amplified using the Nextera XT kit (Illumina Inc., San Diego, CA, USA) and following the manufacturer’s protocol. In brief, one library amplification reaction contained 5 µL of 0.2 ng/µL purified amplicon pool sample, 15 µL tagmentation mix, 5 µL neutralization buffer, 15 µL Nextera PCR mix, and 10 µL index mix (5 µL i7, 5 µL i5). Tagmentation reactions containing the template and the tagmentation mix were incubated at 55°C for 5 minutes and quickly neutralized thereafter. The library amplification reactions were cycled as follows: 72°C for 3 minutes, 95°C for 10 seconds, 55°C for 30 seconds, 72°C for 30 seconds (steps 2–4, 15 cycles), 72°C for 5 minutes, and 10°C hold. Amplified libraries were purified as described above for the amplicon pools with an equal volume (50 µL) of AxyPrep MagPCR cleanup beads and eluted in 50 µL. DNA concentrations of individual libraries were determined using Qubit HS (ThermoFisher Scientific) and normalized to 1 ng/µL in a 25-µL volume. Library pools were constructed by mixing 10 µL of each individual library for a total volume of 320 µL.

The concentration of the library pools was adjusted to 120 pM, and 20 µL of the library pool (10% *phi*X spike) were loaded onto iSeq 100 i1 Reagent v2 (300-cycle) cartridges and sequenced on iSeq instruments (Illumina).

### Bioinformatic analysis

Sequencing data were analyzed using a custom, in-house developed pipeline (https://github.com/BPHL-Molecular/Mycoplasma_target) for read quality control processing to map reads to the *M. genitalium* G37 reference, L43967.2 (NCBI GenBank), and variant calling. FastQC (v0.11.9) and MultiQC were used for quality control. Trimmomatic (v0.39) and BBDuk of bbtools (v38.76) were used to remove Illumina adapter sequences and any *phi*X contamination. BWA (v0.7.17), SAMtools (v1.12), and BCFtools (v1.12) were used for reads mapping, alignment, and variant calls in VCF. BEDTools (v2.18) was used for the intersection of the reference gff file and variant (SNP) VCF file. Additionally, each .bam file was visualized in IGV (Integrative Genomics Viewer, https://igv.org/). Each gene region target was checked for even read coverage, ≥100× mean depth, and overall clean quality. SNPs were also visually confirmed in IGV and compared to those identified using the in-house pipeline. Publicly available *M. genitalium* sequences (11) have been tested by the pipeline used in this study with satisfactory results (data not shown). All SNPs reported in this study had a percentage of alternate alleles ≥20%. Mutations identified by comparing with *M. genitalium* reference strain G37 were cataloged and compared with mutations reported in the literature to be associated with macrolide and/or fluoroquinolone resistance.

## RESULTS

After extraction, amplification, and evaluation by gel electrophoresis, 143 samples were determined to be satisfactory for sequencing. The specimen source did not have great significance, as swabs performed only marginally better than urine in having successful PCR amplifications (54.5% vs 53.8%). A total of 98 out of the above 143 samples (68.5%) yielded satisfactory sequences for *all seven* amplicons, whereas the remaining 45/143 produced satisfactory sequences for some of the amplicons: *n* = 33 failed PCR for one amplicon, *n* = 8 failed PCR for two amplicons, *n* = 1 failed PCR for three amplicons, and *n* = 3 failed PCR for five amplicons. Overall, the best amplified and sequenced locus was *gyrA* (141/143) and the worst amplified was *parE* (118/143 successful). Amplification for 23S rRNA was successful in 137/143 samples, and 7/137 did not sequence successfully. The depth of coverage for a successfully sequenced amplicon was typically >1,000×.

### Macrolide resistance: *rrl* (23S rRNA)

For this locus, satisfactory sequences were obtained for 91% (130/143) of the samples. A total of 82% (106/130) of the samples had at least one mutation associated with macrolide resistance. The mutations observed are listed in *M. genitalium* 23S rRNA numeration and include A2071G (*n* = 38), A2071T (*n* = 1), A2072G (*n* = 61), and A2072C (*n* = 2). The T2199G (*n* = 17) mutation was found both as a single mutation (*n* = 3) and in combination with other strong mutations (A2071G + T2199G, *n* = 2; A2072G + T2199G, *n* = 10). Other combinations that are likely to induce resistance to macrolides included A2071G + A2072C (*n* = 1), A2071G + G2138A (*n* = 1), A2072G + C2097T (*n* = 1), and A2072G + G2217A (*n* = 1). We found two isolates with more than two mutations (A2072G + C2457T + C2479A + C2537G + G2554C) and (G2080A + T2199G + A2298G + A2302C + T2309C + T2314C + A2330G + T2332A). The mutation C2240T (*n* = 1) had uncertain significance in terms of resistance to macrolides. The *rrl* data are summarized in Table 2 and the supplemental data table.

**TABLE 2:** *rrl* (23S rRNA) mutation summary data

Mutations *rrl*	Single nucleotide polymorphisms^*a*^
SNP	**A2071G (38), A2071T (1), A2072C (2), A2072G (61**), G2080A (1), C2097T (1), G2138A (1), **T2199G (17**), G2217A (1), C2240T (1), A2298G (1), A2302C (1), T2309C (1), T2314C (1), A2330G (1), T2332A (1), C2457T (1), C2479A (1), C2537G (1), G2554C (1)

^*a*^ The number of total SNPs is given in parentheses; mutations published to be associated with macrolide resistance are shown in bold.

### Macrolide resistance: *rpl*D (ribosomal protein L4)

For this gene locus, satisfactory sequences were obtained for 94% (135/143) of the samples, and a total of 94 mutations were identified. Nineteen samples harbored one mutation per isolate, and 14 isolates had multiple mutations: 40% (38/94) were amino acid substitutions, and the remaining ones were synonymous mutations. Three samples had an amino acid change, Pro81Ser, that was reported to be associated with macrolide resistance (13). Interestingly, one of the Pro81Ser-positive samples (#34, Table S1) exhibited no 23S rRNA MRMs, and a second one (#64, Table S1) failed to amplify 23S rRNA (no results available). The *rpl*D data are summarized in Table 3 and in the supplemental data table.

**TABLE 3:** *rpl*D (L4) mutation summary data

Mutations *rpl*D	Amino acid substitutions^*a*^
Nonsynonymous	Ala28Pro (3), Lys66Glu (1), His69Arg (10), Asn80Asp (1), **Pro81Ser** (3), Ala114Asp (1), Ala114Val (3), Ala116Val (3), Ala144Val (1), Pro166Ser (1), Asn172Ser (9), Thr204Ala (2)
Silent	Ser50Ser (6), Ala73Ala (1), Gln75Gln (6), Pro81Pro (1), Gly86Gly (5), Leu109Leu (4), Lys118Lys (2), Asn120Asn (4), Leu143Leu (8) Leu146Leu (8), Leu151Leu (1), Leu156Leu (1), Leu169Leu (9)

^*a*^ The number of isolates for each mutation is given in parentheses; mutations published to be associated with macrolide resistance are shown in bold.

### Macrolide resistance: *rpl*V (ribosomal protein L22)

A total of 136 out of 143 samples passed QC for the *rpl*V gene (95%): 48 samples had at least one mutation, and 59 total mutations were observed, of which 10% (6/59) were amino acid substitutions and 90% were silent mutations. None of the nonsynonymous mutations observed have been reported in the literature as being associated with macrolide resistance, but we have identified two missense mutations present in two samples: Gln141STOP (CAA > TAA) and Gln144STOP (CAA > TAA). As the entire L22 protein is 144 amino acids long, we do not know whether these early chain termination mutations affect the protein structure of L22 and have any impact on resistance to macrolides in the isolates carrying them, but it is worth noting that both nonsense mutations also exhibited 23S rRNA mutations: A2071G and T2199G, respectively. Data for *rpl*V are presented in Table 4.

**TABLE 4:** *rpl*V (L22) mutation summary data

Mutations *rpl*V	Amino acid substitutions^*a*^
Nonsynonymous	Ala44Ser (1), Gly93Val (2) Gln139Lys (1), Gln141STOP (1), Gln144STOP (1)
Silent	Arg18Arg (6), Cys21Cys (1), Lys27Lys (9), Ile35Ile (1), Ala54Ala (1), Asn77Asn (24), Ala89Ala (1), Thr97Thr (1) Leu117Leu (8), Leu124Leu (1)

^*a*^ The number of isolates for each mutation is given in parentheses.

### Fluoroquinolone resistance: *gyr*A (DNA gyrase subunit A)

For *gyr*A, satisfactory sequences were obtained for 99% (141/143) samples. Two samples had nonsynonymous *gyr*A mutations: Ile97Val (ATA > GTA) and Ala105Thr (GCT > ACT). While neither of these mutations were reported to be associated with fluroquinolone resistance, both harbored mutations in the *rrl* gene (23S rRNA) associated with macrolide resistance (Ala105Thr + *rrl* T2199G, and Ile97Val+ *rrl* A2071G). We cannot speculate at this time whether these two *gyr*A nonsynonymous mutations are part of multidrug resistance phenotypes. Of note, none of the two samples with *gyr*A amino acid substitutions harbored *par*C mutations, a prerequisite for increased fluoroquinolone MICs, as suggested in references (16) and (17). Three other samples had silent mutations in the *gyr*A locus: Phe89Phe (TTC >TTT, *n* = 1) and Gln106Gln (CAA >CAG, *n* = 2).

### Fluoroquinolone resistance: *gyr*B (DNA gyrase subunit B)

For this genetic determinant, satisfactory sequences were obtained for 94% (135/143) samples. A total of 14 out of 135 samples had mutations, including three silent ones. None of the four types of amino acid substitutions contained in the 11 isolates (Ile495Val *n* = 3, Ser507Asn *n* = 1, Tyr438His *n* = 1, Val538Ile *n* = 6) mutations observed in *gyr*B have been reported in the literature as being associated with fluoroquinolone resistance in *M. genitalium,* but 10 exhibited also *rrl* mutations. As for *gyr*A, none of the samples of the *gyr*B nonsynonymous mutations exhibited *par*C amino acid substitutions. GyrB silent mutations detected were Asp498Asp, Thr513Thr, and Thr524Thr.

### Fluoroquinolone resistance: *par*C (DNA topoisomerase IV, subunit A)

For the *par*C gene, satisfactory sequences were obtained for 90% (129/143) of the samples. The 77 mutations detected were harbored by 58 of the samples: 53 out of 77 mutations were synonymous, and among the 24 true amino acid substitutions, 23 affected amino acid residues were described previously to be involved in fluoroquinolone resistance (Pro62, Asp82, Ser83, and Asp87) and one was not (Ser84Pro). Mutations at Ser83 (*n* = 1) and Asp87 (*n* = 4) are considered to be the most common mutations linked to fluoroquinolone resistance; data for *par*C are summarized in Table 5.

**TABLE 5:** *par*C mutation summary data

Mutations *par*C	Amino acid substitutions^*a*^
Nonsynonymous	**Pro62Ser** (8), **Asp82Asn** (10), **Ser83Ile** (1), Ser84Pro (1), **Asp87Asn** (3), **Asp87Tyr** (1)
Silent	Lys11Lys (12), Val44Val (7), Ile48Ile (1), His78His (31), Pro79Pro (1), Ser93Ser (1)

^*a*^ The number of isolates for each mutation is given in parentheses; mutations published to be associated with fluoroquinolone resistance are shown in bold.

### Fluoroquinolone resistance: *par*E (DNA topoisomerase IV, subunit B)

For the *par*E locus, satisfactory sequences were obtained for 80% (114/143) of the samples, and *par*E was the worst-performing amplicon in our study. The 70 mutations detected were found in 49 of the samples tested. The 36 silent mutations and 34 amino acid substitutions were distributed rather randomly in the genetic sequence we investigated (amino acids 119–509) and affected 32 distinct amino acid residues. None of the mutations observed were reported to be associated with resistance. The data for *par*E are summarized in Table 6.

**TABLE 6:** *par*E mutation summary data

Mutations *par*E	Amino acid substitutions^*a*^
Nonsynonymous	Val133Glu (2), Ile139Val (1), Asp159Glu (5), His163Arg (1), Asp182Asn (1), Ser183Asn (1), Phe224Ser (1), Glu244Asp (1), Asp246An (1), Gly247Glu (1), Val250Leu (1) Ser293Arg (1), Glu344Lys (1), Val353Ile (2) Ala368Ser (1), Ala368Thr (1), Ala368Val (1), Ser396Leu (1), Pro398Arg (2), Pro398Ser (1), Thr413Ile (5), Pro446Ser (1), Glu462Lys (1)
Silent	Leu129Leu (4), Val133Val (3), Gly158Gly (1), Ser162Ser (3), Thr165Thr (1), Ser188Ser (1), Leu202Leu (1), Ser270Ser (3), Lys297Lys (5), Lys343Lys (1), Leu376Leu (5), Gly431Gly (4), Ala463Ala (4)

^*a*^ The number of isolates for each mutation is given in parentheses.

## DISCUSSION

This study explored the feasibility of determining drug resistance (macrolides and fluoroquinolones) using molecular methods (DNA sequencing) in *M. genitalium*-positive samples. This culture-independent method used a partial multiplex design (four PCRs per sample) and seven primer sets to sequence the regions of the *M. genitalium* genome known to be associated with antimicrobial resistance, covering 0.77% of the entire genome. The success rate for sequencing of PCR-amplified samples was >90% for all but one of the amplicons (*par*E), but future research and a primer re-design might improve the *par*E numbers. It is noteworthy that valid results for the two major determinants (*rrl*-macrolides and *par*C-fluoroquinolones) were observed in 91% and 90.2% of the samples, respectively. A previous study using tNGS for *M. genitalium* (11) sequenced only three resistance amplicons (23S rRNA, *gyr*A, and *par*C), and this might be sufficient in some cases to characterize drug resistance. This work had a maximalist approach (most of the samples included had all seven amplicons present), and we believe that valuable drug-resistance information can be obtained for samples not having the entire panel successfully amplified. However, at least one sample exhibited a *rpl*D mutation (Pro81Ser) and had no *rrl* SNPs; by using only 23S rRNA sequencing to determine resistance to macrolides, this sample would have been probably deemed susceptible.

This study revealed an unusually high rate of resistance to macrolides in the state of Florida (82% of all isolates tested had mutations published to cause macrolide resistance). This is significantly higher than what was described in other studies (18–20), but a study in gay sex workers in China described similar rates of resistance, 83% (21). In our population, acquired resistance is a tempting hypothesis, but as this study included de-identified specimens, it is not possible for us to know whether this high level of macrolide resistance is due to previous treatment regimens. *par*C is the most common locus cited in the literature as responsible for fluoroquinolone resistance in *M. genitalium*, and a certain number of mutations matching the ones published in the literature to be FQ-resistant were identified in our population as well. Remarkably, 23 out of 24 of the nonsynonymous *par*C mutations affect amino acid residues previously linked to FQ resistance. A novel amino acid substitution belonging to the *par*C QRDR (TCC > CCC, Ser84Pro) has been identified; its role in drug resistance needs to be established.

As in other studies (22, 23), the *gyr*A locus in *M. genitalium* is remarkably well conserved in our population also (e.g., only two samples have exhibited amino acid substitutions in *gyr*A, both with uncertain significance), and none of the 10 nonsynonymous mutations in *gyr*B identified in this study were previously published. Thus, in our setting, as described in other works, *parC* appears to be the major determinant for fluoroquinolone resistance, while the contribution of gyrase loci may be considered minor.

Seventeen isolates were determined to exhibit multidrug resistance (macrolide and fluoroquinolones), which can have implications for designing effective treatment regimens for patients infected with these kinds of strains. While successful treatments for these kinds of cases with minocycline have been described (24), it is important for clinicians to have this information as soon as possible.

It is reasonable to believe that not all the nonsynonymous mutations identified in this work are relevant for drug resistance in *M. genitalium*, but some of them may impact protein structures in a significant manner, so as to lead to protein variants that are unproperly folded and hence ineffective. This would be appealing to test *in silico*, but it was not the object of this study. Some of the amino acid substitutions identified in our study may describe (in combination or not with silent mutations) some specific lineages. The *par*E locus especially proved to be extraordinarily variable, with 70 mutations detected in total, more than half of them being silent. At this moment, we do not know either how many of the large number of silent mutations detected with our method (*n* = 204, in all loci) represent true lineage markers or if the variability of *rpl*D and *rpl*V can be used for genotyping of this bacteria. According to (1), a unified view regarding *M. genitalium* genotyping has not crystallized yet, but we hope that the accumulation of more genetic data will provide more clarity soon.

As with reference (11), we believe that targeted sequencing is a good, dependable (despite some limitations), scalable, and relatively high-throughput, culture-independent approach to detect drug resistance-associated mutations in *M. genitalium*. With next-generation sequencing instruments being now almost ubiquitous in US public health laboratories, we are confident that this kind of approach will find its utility in evaluating drug resistance profiles of fastidious microorganisms.

### Limitations of the study

Only 54% of the extracted samples yielded amplicons satisfactory for sequencing, and the Hologic media may not be the most suitable for *M. genitalium* preservation. Thus, additional research is required to determine whether the success rate is dependent on the transport media.

As this study involved testing de-identified samples, no information was collected regarding patient history, including symptoms, diagnosis, or treatment history. The novel mutations uncovered here remain of uncertain significance, as we do not have the possibility of culturing this microorganism in our laboratory, and the Hologic transport medium does not allow recovery of viable bacteria. Therefore, no inferences can be made regarding the unpublished nonsynonymous mutations identified here and eventual treatment failures/persistent infections, indicative of drug resistance.

The research described here is not a validation study to compare or benchmark our approach to other methods, but a proof of concept meant to be used as a starting point for laboratories interested in implementing a similar or simplified method.

### Software license

The custom pipeline (https://github.com/BPHL-Molecular/Mycoplasma_target) is available under open-source license MIT.

## SUPPLEMENTAL MATERIAL

Supplemental table spectrum.03845-23-s0001.docx Sample data by primer.

ASM does not own the copyrights to Supplemental Material that may be linked to, or accessed through, an article. The authors have granted ASM a non-exclusive, world-wide license to publish the Supplemental Material files. Please contact the corresponding author directly for reuse.
