# Supplementary material for: Detection of resistance to macrolides and fluoroquinolones in Mycoplasma genitalium by targeted next-generation sequencing
Source: Microbiol Spectr. 2024 Feb 13;12(3):e03845-23. doi: 10.1128/spectrum.03845-23 (PMC10913745; doi:10.1128/spectrum.03845-23)
Supplement: Supplemental table — Sample data by primer. [file spectrum.03845-23-s0001.docx]

Calin B. Chiribau et al., Detection of Resistance to Macrolides and Fluoroquinolones in *Mycoplasma genitalium* by Targeted Next Generation Sequencing (Supplemental data)

| **Sample \ Locus** | ***rrl*** | ***gyrA*** | ***gyrB*** | ***rplD*** | ***rplV*** | ***parC*** | ***parE*** |
| --- | --- | --- | --- | --- | --- | --- | --- |
| 1 | A2071G | NO | NO | NO | NO | His78His | NO |
| 2 | A2072G | NO | NO | NO | NO | NO | Val353Ile |
| 3 | FAIL PCR | NO | FAIL PCR | NO | FAIL PCR | FAIL PCR | FAIL PCR |
| 4 | FAIL SEQ | NO | FAIL PCR | FAIL PCR | FAIL PCR | FAIL PCR | FAIL PCR |
| 5 | A2072G | FAIL PCR | FAIL PCR | NO | Leu124Leu | Asp82Asn | NO |
| 6 | NO | NO | NO | Ser50Ser, Gln75Gln, Gly86Gly | Leu117Leu | Lys11Lys, Val44Val, Pro62Ser | NO |
| 7 | A2072G, T2199G | NO | NO | His69Arg | NO | NO | FAIL PCR |
| 8 | A2072G | NO | Val538Ile | NO | Asn77Asn | NO | NO |
| 9 | A2071G | NO | NO | NO | NO | His78His | NO |
| 10 | A2071G | NO | FAIL PCR | NO | NO | FAIL PCR | NO |
| 11 | A2071G | NO | NO | NO | NO | His78His, Asp87Tyr | Ser396Leu |
| 12 | A2072C, T2199G | NO | NO | NO | NO | NO | NO |
| 13 | A2071G | NO | Val538Ile | NO | Asn77Asn, Gly93Val | NO | Ser270Ser |
| 14 | A2072G | NO | Val538Ile | NO | Asn77Asn | NO | Ser183Asn, Glu244Asp |
| 15 | A2072G | NO | NO | NO | NO | NO | NO |
| 16 | A2072G | NO | NO | His69Arg | Asn77Asn | NO | Ala463Ala |
| 17 | NO | NO | Thr513Thr | Ala28Pro | NO | FAIL PCR | FAIL PCR |
| 18 | FAIL SEQ | NO | FAIL PCR | FAIL PCR | FAIL PCR | FAIL PCR | FAIL PCR |
| 19 | NO | NO | NO | Pro81Pro | NO | His78His | NO |
| 20 | A2072G | NO | NO | NO | NO | NO | Leu376Leu, Glu462Lys |
| 21 | A2072G | NO | Tyr438His | NO | NO | NO | Thr413Ile |
| 22 | NO | NO | NO | Ser50Ser, Gln75Gln, Gly86Gly, Ala144Val, Ala114Asp, Ala116Val, Asn120Asn, Leu169Leu, Asn172Ser | Leu117Leu | NO | Pro398Arg |
| 23 | G2080A, T2199G, A2298G, A2302C, T2309C, T2314C, A2330G, T2332A | NO | NO | NO | FAIL PCR | NO | NO |
| 24 | NO | NO | NO | Leu151Leu | NO | NO | Ile139Val |
| 25 | A2071G, G2138A | NO | NO | NO | NO | His78His | Gly431Gly |
| 26 | A2071G | NO | NO | NO | NO | His78His | NO |
| 27 | FAIL PCR | NO | NO | NO | FAIL PCR | NO | Asp182Asn |
| 28 | A2071G | NO | Val538Ile | NO | Asn77Asn | NO | Ser270Ser |
| 29 | NO | NO | NO | NO | NO | NO | NO |
| 30 | A2071G | NO | FAIL PCR | NO | NO | Asp87Asn | FAIL PCR |
| 31 | A2072G | NO | NO | NO | NO | Asp82Asn | NO |
| 32 | A2072G | NO | NO | Lys118Lys | NO | His78His | NO |
| 33 | A2071G | NO | NO | NO | NO | His78His | Ala368Ser |
| 34 | NO | NO | NO | Ala28Pro, Pro81Ser | Asn77Asn | NO | NO |
| 35 | A2072G | NO | NO | NO | NO | Asp82Asn | NO |
| 36 | A2071G, T2199G | NO | NO | NO | Leu117Leu | NO | NO |
| 37 | A2071T | NO | NO | FAIL PCR | NO | FAIL PCR | NO |
| 38 | NO | NO | NO | Ala28Pro | NO | FAIL PCR | NO |
| 39 | NO | NO | Ser507Asn | Leu109Leu, Leu143Leu, Leu146Leu, Leu169Leu, Asn172Ser, Asn172Ser | NO | NO | NO |
| 40 | A2072G | NO | NO | His69Arg | Asn77Asn, Thr97Thr | NO | Ala463Ala |
| 41 | A2071G, A2072C | NO | Ile495Val | NO | NO | His78His | Pro446Ser |
| 42 | A2072G, T2199G | NO | NO | NO | NO | His78His | NO |
| 43 | NO | NO | NO | Ser50Ser, Gln75Gln | Leu117Leu | NO | NO |
| 44 | A2072G, T2199G | NO | NO | NO | Asn77Asn | NO | Pro398Ser |
| 45 | A2072G | NO | NO | NO | NO | NO | Val353Ile |
| 46 | NO | NO | NO | NO | NO | Asp82Asn | FAIL PCR |
| 47 | A2072G | NO | NO | NO | NO | NO | NO |
| 48 | A2071G | NO | NO | NO | NO | His78His | Ser162Ser |
| 49 | FAIL SEQ | NO | NO | FAIL PCR | NO | NO | FAIL SEQ |
| 50 | A2072G | NO | NO | NO | NO | His78His | NO |
| 51 | A2072G, T2199G | NO | NO | NO | NO | NO | Thr165Thr |
| 52 | A2072G | NO | Val538Ile | NO | Asn77Asn | NO | NO |
| 53 | A2071G | NO | NO | NO | Leu117Leu | FAIL PCR | His163Arg |
| 54 | FAIL PCR | NO | NO | His69Arg | NO | FAIL PCR | FAIL SEQ |
| 55 | A2072G, C2457T, C2479A, C2537G, G2554C | NO | NO | NO | NO | NO | FAIL PCR |
| 56 | A2072G | NO | NO | NO | Gly93Val | Ser84Pro | Thr413Ile |
| 57 | A2072G | NO | NO | NO | NO | Asp82Asn | NO |
| 58 | A2072G | NO | NO | Leu109Leu, Leu143Leu, Leu146Leu, Leu196Leu, Asn172Ser | Lys27Lys | NO | NO |
| 59 | A2072G | NO | NO | NO | Asn77Asn | Lys11Lys | Leu129Leu, Val133Glu, Asp159Glu, Lys297Lys |
| 60 | A2072G, T2199G | NO | NO | NO | NO | Ser83Ile | NO |
| 61 | A2072G | NO | NO | NO | NO | Lys11Lys, Asp87Asn | Leu129Leu, Val133Glu, Gly158Gly, Asp159Glu, Lys297Lys, Glu344Lys |
| 62 | A2071G | NO | NO | Pro166Ser | NO | NO | Ala368Thr |
| 63 | T2199G | Ala105Thr | NO | NO | Asn77Asn | NO | NO |
| 64 | FAIL PCR | NO | NO | Pro81Ser | Asn77Asn | Lys11Lys, Val44Val, Pro62Ser | NO |
| 65 | C2240T | NO | NO | NO | NO | Pro62Ser | FAIL PCR |
| 66 | NO | Phe89Phe | NO | Ala114Val, Ala116Val, Asn120Asn, Leu143Leu, Leu146Leu, Leu169Leu, Asn172Ser | NO | NO | Leu129Leu, Val133Val, Asp159Glu, Leu202Leu, Phe224Ser |
| 67 | A2072G | NO | NO | NO | NO | Asp82Asn | NO |
| 68 | A2072G | NO | NO | His69Arg | Asn77Asn | His78His | NO |
| 69 | FAIL SEQ | NO | NO | NO | NO | FAIL PCR | NO |
| 70 | NO | NO | NO | NO | Arg18Arg, Lys27Lys | FAIL PCR | FAIL PCR |
| 71 | NO | NO | NO | Ser50Ser, Gln75Gln, Gly86Gly, Ala114Val, Ala116Val, Asn120Asn, Leu143Leu, Leu146Leu | Leu117Leu | NO | Pro398Arg |
| 72 | A2071G | NO | Ile495Val | Lys66Glu, His69Arg, Leu109Leu, Leu143Leu, Leu146Leu, Leu156Leu, Leu169Leu, Asn172Ser | Lys27Lys | NO | FAIL PCR |
| 73 | A2072G | NO | NO | His69Arg | Asn77Asn | NO | Ala463Ala |
| 74 | A2072G, G2217A | NO | NO | NO | Ala44Ser | NO | NO |
| 75 | A2071G | NO | FAIL PCR | NO | Arg18Arg, Lys27Lys | FAIL SEQ | NO |
| 76 | A2071G | NO | NO | His69Arg | FAIL PCR | NO | Ala463Ala |
| 77 | NO | NO | NO | NO | Arg18Arg, Lys27Lys | NO | Leu376Leu |
| 78 | FAIL PCR | NO | NO | NO | Asn77Asn | Ile48Ile, Pro79Pro | NO |
| 79 | A2071G | NO | NO | NO | NO | His78His | NO |
| 80 | A2072G | NO | NO | NO | NO | NO | FAIL PCR |
| 81 | A2071G, T2199G | NO | NO | Pro81Ser | Asn77Asn | Lys11Lys, Val44Val, Pro62Ser | NO |
| 82 | FAIL PCR | NO | NO | NO | NO | His78His | Ala368Val |
| 83 | A2072G | NO | NO | NO | NO | NO | NO |
| 84 | FAIL SEQ | NO | FAIL SEQ | FAIL SEQ | FAIL SEQ | FAIL PCR | FAIL SEQ |
| 85 | A2072G | NO | NO | NO | NO | His78His | Ser188Ser |
| 86 | A2071G | NO | NO | NO | Arg18Arg, Lys27Lys | NO | Leu376Leu |
| 87 | NO | NO | NO | NO | Asn77Asn | Lys11Lys | Leu129Leu, Val133Val, Asp159Glu, Lys297Lys |
| 88 | T2199G | NO | NO | NO | Asn77Asn, Glu141STOP | NO | FAIL PCR |
| 89 | NO | NO | NO | FAIL PCR | Cys21Cys, Asn77Asn | Lys11Lys, Val44Val, Pro62Ser | NO |
| 90 | A2072G | NO | Thr524Thr | Leu109Leu, Leu143Leu, Leu146Leu, Leu169Leu, Asn172Ser | Lys27Lys | NO | NO |
| 91 | FAIL SEQ | NO | NO | NO | NO | NO | FAIL PCR |
| 92 | A2071G | NO | NO | NO | NO | His78His | FAIL PCR |
| 93 | A2072G | NO | NO | NO | NO | NO | FAIL PCR |
| 94 | A2071G | NO | NO | NO | NO | His78His | Lys343Lys |
| 95 | A2071G | NO | NO | NO | NO | His78His | FAIL PCR |
| 96 | A2071G | NO | Ile495Val | NO | NO | His78His | NO |
| 97 | A2072G | NO | NO | NO | NO | NO | FAIL PCR |
| 98 | A2072G | NO | NO | NO | Ile35Ile | NO | NO |
| 99 | NO | NO | Asp498Asp | FAIL PCR | Asn77Asn | Lys11Lys | Leu129Leu, Val133Val, Asp159Glu, Gly247Glu, Lys297Lys |
| 100 | A2071G | NO | NO | NO | NO | His78His | Gly431Gly |
| 101 | A2071G | NO | NO | NO | Gln139Lys | His78His | Gly431Gly |
| 102 | A2072G | NO | NO | NO | NO | NO | NO |
| 103 | A2071G | FAIL PCR | NO | NO | Ala54Ala | NO | FAIL PCR |
| 104 | A2072G | NO | NO | NO | NO | NO | Thr413Ile |
| 105 | A2072G | NO | NO | NO | NO | Asp82Asn | FAIL PCR |
| 106 | FAIL SEQ | NO | NO | Asn80Asp, Thr204Ala | Arg18Arg, Lys27Lys | NO | FAIL PCR |
| 107 | A2072G | NO | NO | NO | NO | Asp82Asn | FAIL PCR |
| 108 | NO | NO | NO | Ser50Ser, Gln75Gln, Gly86Gly, Leu169Leu | Leu117Leu | Lys11Lys, Val44Val, Pro62Ser | NO |
| 109 | NO | NO | NO | NO | NO | NO | NO |
| 110 | A2071G | NO | NO | NO | NO | His78His | FAIL PCR |
| 111 | NO | NO | NO | NO | NO | His78His | Asp246Asn |
| 112 | A2071G | NO | NO | NO | NO | NO | NO |
| 113 | A2072G | NO | NO | Lys118Lys | NO | NO | FAIL PCR |
| 114 | A2072G | NO | NO | NO | NO | NO | Leu376Leu |
| 115 | A2072G | NO | NO | NO | Ala89Ala | NO | Val250Leu |
| 116 | A2071G | Ile97Val | NO | Ala73Ala, Leu143Leu, Leu146Leu, Asn172Ser | Asn77Asn, Gln144STOP | NO | NO |
| 117 | NO | NO | NO | NO | NO | NO | NO |
| 118 | A2072G | NO | NO | NO | NO | NO | NO |
| 119 | A2071G | NO | NO | NO | NO | His78His | Gly431Gly |
| 120 | A2072G, T2199G | Gln106Gln | NO | NO | Asn77Asn | NO | NO |
| 121 | A2071G | NO | NO | NO | NO | His78His | NO |
| 122 | A2072G, T2199G | NO | NO | His69Arg | NO | NO | NO |
| 123 | NO | NO | NO | NO | NO | His78His | NO |
| 124 | A2072G | NO | NO | NO | NO | NO | NO |
| 125 | A2072G, C2097T | NO | NO | NO | NO | Asp82Asn | Thr413Ile |
| 126 | A2072G | NO | NO | NO | NO | NO | Leu376Leu |
| 127 | T2199G | NO | NO | NO | NO | Ser93Ser | NO |
| 128 | NO | NO | NO | Ser50Ser, Gln75Gln, Gly86Gly, Ala114Val, Asn120Asn, Leu143Leu, Leu146Leu, Leu196Leu, Asn172Ser | Leu117Leu | FAIL PCR | Ser293Arg |
| 129 | A2071G | NO | NO | NO | NO | NO | Ser162Ser |
| 130 | A2072G | NO | NO | Thr204Ala | Arg18Arg, Lys27Lys | NO | NO |
| 131 | A2072G | NO | NO | NO | NO | Lys11Lys, Asp87Asn | FAIL PCR |
| 132 | A2072G | NO | NO | His69Arg | NO | Lys11Lys, Val44Val, Pro62Ser | NO |
| 133 | A2072G | NO | NO | NO | NO | Asp82Asn | NO |
| 134 | A2072G | NO | Val538Ile | NO | Asn77Asn | NO | Ser270Ser |
| 135 | A2071G | NO | NO | NO | NO | His78His | NO |
| 136 | A2072G | NO | NO | NO | NO | NO | Thr413Ile |
| 137 | A2072G | NO | NO | NO | NO | NO | NO |
| 138 | A2072G, T2199G | NO | NO | NO | NO | NO | NO |
| 139 | A2072G, T2199G | NO | NO | NO | NO | Lys11Lys, Val44Val, Pro62Ser, His78His | FAIL PCR |
| 140 | A2071G | NO | NO | NO | NO | His78His | Ser162Ser |
| 141 | A2071G | NO | NO | NO | NO | NO | NO |
| 142 | A2071G | NO | NO | NO | NO | His78His | NO |
| 143 | A2072G, T2199G | Gln106Gln | NO | NO | Asn77Asn | NO | NO |
